# Supplementary material for: Impact of Lockdown Measures on Health Outcomes of Adults with Type 2 Diabetes Mellitus in Bangladesh
Source: Healthcare (Basel). 2023 Apr 21;11(8):1191. doi: 10.3390/healthcare11081191 (PMC10137871; doi:10.3390/healthcare11081191)
Supplement: Supplementary file 1 [file healthcare-11-01191-s001.zip › healthcare-2320197-supplementary.pdf]

Supplementary Tables

Table S1 - Key clinical data sets included in the CRFs and potential targets for patients with T2DM.

Clinical data	Nature and targets
Glycemic Control (Extent including any hypoglycemic episodes)	• HbA1c target of 7.0% (53 mmol/mol) up to 7.9% among people with T2DM will reduce the risk of macrovascular and microvascular disease; a target of 6.5% to 6.03% (48 mmol/mol) is more appropriate at diagnosis [30,54, 66-70].<br>• Alternatively, FPG levels ≥ 126 mg/dL down to 104mg/dl for early diagnosis [67,68].<br>• PPG target <10.0mol/l, with clinical studies showing targeting PPG can further improve glycemic control [50,69]
BMI [50,71]	• Calculated as weight in Kg/ height in m^2<br>• A lower BMI cut-off value was used to define obesity for Asian population: Normal range: 18.50 – 22.99; Overweight: ≥23.00; Obese: ≥27.50<br>• This difference from the American population reflects differences in body fat and musculature, with Asian patients tending to have more body fat, especially abdominal fat. Consequently, for a given BMI, Asian people tend to have higher weight related disease risks at lower BMIs than their Americans counterparts – leading to lower BMI cut-off levels
Medicines prescribed for glycemic control [50,51, 71, 72]	• Oral treatments including Sulphonylureas (Gliclazide, Glimepiride) and Metformin, DPPiv I, Glinides, Voglibose and SGLT2i<br>• Injectable: Insulins including regular, intermediate, pre-mixed insulins and long-acting insulin analogues as well as GLP1 RA
Blood Pressure [50, 71, 73-75]	• BP targets in guidelines vary between < 130/80mmHg and <140/90 mmHg; however, a target <140/90 mmHg predominates<br>• Data on the range of different medicines and classes will be collected including diuretics, beta-blockers, calcium channel blockers as well as medicines acting on the angiotensin system including angiotensin receptor blockers<br>• Typically, medicines acting on the angiotensin system are also prescribed to reduce diabetic nephropathy
Lipid profiles [50, 69, 71, 76-78]	Targets in Bangladesh with lipid lowering medicines known to decrease subsequent cardiovascular events in patients with T2DM included:<br>• Total Cholesterol (TC) < 200mg/dl<br>• High density lipoprotein (HDL) > 40 mg/dl in males and > 50 mg/dl in females<br>• Low density lipoprotein (LDL) < 100mg/dl<br>• Triglyceride (TG) < 150 mg/dl<br>• Alternatively: TC <4.5 mmol/l, LDL-C <1.8 mmol/l, HDL-C <1.0 for males and <1.3 mmol/l for female and triglycerides <1.7 mmol/l
Serum creatinine and urinary albumin [73, 74, 79-83]	• Urinary albumin: creatinine ratio (ACR): 3-30 mg/mmol OR 30-300mg/g in a spot urine sample (preferably early morning)<br>• Albuminuria (present when UACR > 30 mg/g) - marker for chronic kidney disease<br>• Macroalbuminuria if albumin is present in routine and microscopic examination of urine
Microvascular complications [24, 50, 84, 85]	Includes retinopathy, nephropathy and neuropathy with a diagnosis typically based on clinical experience
Macrovascular complications [24, 75, 84, 86-88]	• Includes cardiovascular and cerebrovascular disease as well as peripheral arterial disease and diabetic foot ulcerations<br>• Anticoagulants are typically prescribed along with lipid lowering therapies to reduce CV events<br>• Diagnosis typically based on clinical experience

Hospitalizations [30]	• History of hospitalizations<br>• Rationale - recorded in patients' discharge notes from the hospital
Any other co-morbidities/ complications [89, 90]	This could include asthma and chronic obstructive pulmonary disease (COPD) as well as thyroid disorders

Table S2 - Treatments prescribed to manage patients' blood sugar levels.

**Diabetes Medicine**	**Visit-1**	**Visit-2**	**Visit-3**	**Visit-4**	**Visit-5**
Metformin					
No	8 (1.2%)	5 (0.7%)	56 (7.7%)	27 (3.9%)	92 (12.6%)
Yes	40 (5.8%)	43 (6.3%)	101 (13.8%)	305 (44.3%)	595 (81.5%)
NR	640 (93.0%)	640 (93.0%)	573 (78.5%)	356 (51.7%)	43 (5.9%)
Gliclazide					
No	75 (10.3%)	76 (10.4%)	131 (17.9%)	307 (42.1%)	571 (78.2%)
Yes	15 (2.1%)	14 (1.90%)	26(3.6%)	67 (9.2%)	116 (15.9%)
NR	640 (93.0%)	640 (87.7%)	573 (78.5%)	356 (48.8%)	43 (5.9%)
Glimepiride					
No	47 (6.8%)	48 (7.0%)	111 (16.1%)	314 (45.6%)	614 (89.2%)
Yes	1 (0.1%)	0	4 (0.6%)	18 (2.6%)	31 (4.5%)
NR	640 (93.0%)	640 (7.0%)	573 (83.3%)	356 (51.7%)	43 (6.3%)
A Glinide					
No	89 (12.2%)	90 (12.3%)	152 (20.8%)	355 (48.6%)	650 (89.0%)
Yes	1 (0.10%)	0	5 (0.70%)	19 (2.60%)	37 (5.10%)
NR	640 (87.7%)	640 (87.7%)	573 (78.5%)	356 (48.8%)	43 (5.90%)
DPP4i					
No	38 (5.5%)	32 (4.7%)	72 (10.5%)	177 (25.7%)	343 (49.9%)
Yes	10 (1.5%)	16 (2.3%)	43 (6.3%)	155 (22.5%)	302 (43.9%)
NR	640 (93.0%)	640 (93.0%)	356 (51.7%)	356 (51.7%)	43 (6.3%)
Voglibose					
No	90 (12.3%)	90 (12.3%)	156 21.4%)	373 (51.1%)	680 (93.2%)
Yes	0	0	1(0.10%)	1(0.1%)	7(1.0%)
NR	640 (87.7%)	640 (87.7%)	573 (78.5%)	356 (51.7%)	43 (5.9%)
SGLT2i					
No	80 (11.0%)	74 (10.1%)	114 (15.6%)	219 (30.0%)	385 (52.7%)
Yes	10 (1.4%)	16 (2.20%)	43 (5.90%)	155 (21.2%)	302 (41.4%)
NR	640 (87.7%)	640 (7.0%)	573 (78.5%)	356 (48.8%)	43 (5.9%)
Regular use of insulin					
No	53 (7.30%)	53 (7.30%)	71 (9.7%)	162 (22.2%)	208 (28.5%)
Yes	6 (0.80%)	9 (1.2%)	26 (3.6%)	92 (12.6%)	278 (38.1%)
NR	671 (91.9%)	668 (91.5%)	633 (86.7%)	476 (65.2%)	244 (33.4%)

NB: NR = Not Recorded.

Table S3 - Details of injectable anti-diabetic medicines prescribed including insulins

	Visit-1	Visit-2	Visit-3	Visit-4	Visit-5
Rapid acting analog					
No	59 (8.1%)	62 (8.5%)	905 (13.0%)	246 (33.7%)	472 (64.7%)
Yes	1 (0.1%)	0	1 (0.1%)	8 (1.1%)	13 (1.8%)
NR	670 (91.8%)	668 (91.5%)	634 (86.8%)	476 (65.2%)	245 (33.6%)
Intermediate					
No	56 (7.7%)	57 (7.8%)	83 (11.4%)	208 (28.5%)	303 (41.5%)
Yes	4 (0.5%)	5 (0.7%)	13 (1.8%)	47 (6.4%)	182 (24.9%)
NR	670 (91.8%)	668 (91.5%)	634 (86.8%)	475 (65.1%)	245 (33.6%)
Long-acting analogs					
No	59 (8.1%)	61 (8.4%)	87 (11.9%)	216 (29.6%)	412 (56.4%)
Yes	-	1 (0.1%)	9 (1.2%)	39 (5.3%)	73 (10.0%)
NR	671 (91.9%)	668 (97.1%)	634 (86.8%)	475 (65.1%)	245 (33.6%)
Premixed 30/70					
No	49 (6.7%)	51 (7.0%)	51 (7.0%)	164 (22.5%)	347 (47.5%)
Yes	10 (1.4%)	11 (1.5%)	11 (1.5%)	92 (12.6%)	138 (18.9%)
NR	671 (91.9%)	668 (91.5%)	668 (91.5%)	474 (64.9%)	245 (33.6%)
Premix analog 30/70					
No	59 (8.1%)	62 (8.5%)	91 (12.5%)	228 (31.2%)	445 (61.0%)
Yes	-	-	5 (0.7%)	27 (3.7%)	40 (5.5%)
NR	671 (91.9%)	668 (91.5%)	634 (86.8%)	475 (65.1%)	245 (33.6%)
Premix analog 25/75					
No	17 (2.5%)	20 (2.9%)	54 (7.8%)	211 (30.7%)	440 (64.0%)
Yes	-	-	-	2 (0.3%)	3 (0.40%)
NR	671 (97.5%)	668 (97.1%)	634 (92.2%)	475 (69.0%)	245 (35.6%)
Premixed 50/50					
No	59 (8.1%)	62 (8.5%)	95 (13.0%)	252 (34.5%)	479 (65.6%)
Yes	-	-	-	3 (0.40%)	6 (0.80%)
NR	671 (91.9%)	668 (91.5%)	634 (86.8%)	475 (65.1%)	245 (33.6%)
GLP1-RA					
No	59 (8.1%)	62 (8.5%)	95 (13.0%)	247 (33.8%)	464 (63.6%)
Yes	-	-	1 (0.10%)	8 (1.1%)	19 (2.6%)
NR	671 (91.9%)	668 (91.5%)	634 (86.8%)	475 (65.1%)	247 (33.8%)

NB: NR = Not Recorded

Table S4 - Details of anti-hypertensives, anticoagulants and GTN prescribed.

	**Visit-1**	**Visit-2**	**Visit-3**	**Visit-4**	**Visit-5**
**Angiotensin Receptor Blockers**					
Losartan 25/50/100					
No	70 (9.6%)	72 (9.9%)	112 (15.3%)	251 (34.4%)	424 (58.1%)
Yes	9 (1.2%)	12 (1.6%)	21 (2.9%)	68 (9.3%)	112 (15.3%)
NR	651 (89.2%)	646 (88.5%)	597 (81.8%)	411 (56.3%)	194 (26.6%)
Olmesartan 10/20/40					
No	64 (8.8%)	67 (9.2%)	85 (11.6%)	155 (21.2%)	244 (33.3%)
Yes	14 (1.9%)	16 (2.2%)	48 (6.6%)	164 (22.5%)	292 (40.0%)
NR	652 (89.3%)	647 (88.6%)	597 (81.8%)	411 (56.3%)	194 (26.6%)
Valsartan 80/160					
No	79 (10.8%)	83 (11.4%)	133 (18.2%)	319 (43.7%)	533 (73.0%)
Yes	-	-	-	-	3 (0.40%)
NR	651 (89.2%)	647 (88.6%)	597 (81.8%)	411 (56.3%)	194 (26.6%)
Telmisartan 40/80					
No	76 (10.4%)	81 (11.1%)	131 (17.9%)	306 (41.9%)	500 (68.5%)
Yes	3 (0.4%)	2 (0.3%)	2 (0.3%)	13 (1.8%)	36 (4.9%)
NR	651 (89.2%)	647 (88.6%)	597 (81.8%)	411 (56.3%)	194 (26.6%)
Azilsartan					
No	79 (10.8%)	83 (11.4%)	133 (18.2%)	319 (43.7%)	536 (73.4%)
Yes	-	-	-	-	-
NR	651 (89.2%)	647 (88.6%)	597 (81.8%)	411 (56.3%)	194 (26.6%)
Irbesartan					
No	79 (10.8%)	83 (11.4%)	133 (18.2%)	319 (43.7%)	536 (73.4%)
Yes	-	-	-	-	-
NR	651 (89.2%)	647 (88.6%)	597 (81.8%)	411 (56.3%)	194 (26.6%)
**Angiotensin Converting Enzyme Inhibitors**					
Enalapril 5/10mg					
No	79 (10.8%)	83 (11.4%)	133 (18.2%)	319 (43.7%)	536 (73.4%)
Yes	-	-	-	-	-
NR	651 (89.2%)	647 (88.6%)	597 (81.8%)	411 (56.3%)	194 (26.6%)
Lisinopril					
No	37 (5.4%)	41 (6.0%)	91 (13.2%)	277 (40.3%)	536 (73.4%)
Yes	-	-	-	-	-
NR	651 (94.6%)	647 (94.0%)	597 (86.8%)	411 (59.7%)	194 (28.2%)
Captopril					
No	79 (10.8%)	83 (11.4%)	133 (18.2%)	319 (43.7%)	536 (73.4%)
Yes	-	-	-	-	-
NR	651 (89.2%)	647 (88.6%)	597 (81.8%)	411 (56.3%)	194 (26.6%)
**Calcium Channel Blockers**					

Amlodipine 5/10					
No	67 (9.2%)	105 (14.4%)	63 (9.2%)	266 (36.4%)	443 (60.7%)
Yes	15 (2.1%)	15 (2.1%)	28 (4.1%)	53 (7.3%)	93 (12.7%)
NR	647 (88.6%)	647 (88.6%)	597 (86.8%)	411 (56.3%)	194 (26.6%)
Levamlodipine					
No	79 (10.8%)	83 (11.4%)	133 (18.2%)	319 (43.7%)	536 (73.4%)
Yes	-	-	-	-	-
NR	651 (89.2%)	647 (88.6%)	597 (81.8%)	411 (56.3%)	194 (26.6%)
Cilnidipine 5/10					
No	79 (10.8%)	81 (11.1%)	130 (17.8%)	304 (41.6%)	500 (68.5%)
Yes	-	2 (0.30%)	3 (0.40%)	15 (2.1%)	36 (4.9%)
NR	651 (89.2%)	647 (81.8%)	597 (81.8%)	411 (56.3%)	194 (26.6%)
Lercanidipine					
No	79 (10.8%)	83 (11.4%)	133 (18.1%)	318 (43.6%)	534 (73.2%)
Yes	-	-	-	11 (0.10%)	2 (0.30%)
NR	651 (89.2%)	647 (88.6%)	597 (81.8%)	411 (56.3%)	194 (26.6%)
**β-blockers**					
Bisoprolol 2.5/5					
No	70 (9.6%)	73 (10.0%)	109 (14.9%)	233 (31.9%)	354 (48.5%)
Yes	9 (1.2%)	10 (1.4%)	24 (3.3%)	86 (11.8%)	182 (24.9%)
NR	651 (89.2%)	647 (88.6%)	597 (81.8%)	411 (56.3%)	194 (26.6%)
Metoprolol 25/50 100					
No	78 (10.7%)	82 (11.2%)	128 (17.5%)	304 (41.6%)	510 (69.9%)
Yes	1 (0.10%)	1 (0.10%)	5 (0.7%)	15 (2.10%)	26 (3.6%)
NR	651 (89.2%)	647 (88.6%)	597 (81.8%)	411 (56.3%)	194 (26.6%)
Nebivolol					
No	77 (10.5%)	82 (11.2%)	132 (18.1%)	317 (43.4%)	533 (73.0%)
Yes	2 (0.3%)	1 (0.10%)	1 (0.1%)	2 (0.3%)	3 (0.40%)
NR	651 (89.2%)	647 (88.6%)	597 (81.8%)	411 (56.3%)	194 (26.6%)
**α-blockers**					
Prazosin					
No	79 (10.8%)	83 (11.4%)	132 (18.1%)	316 (43.3%)	523 (71.6%)
Yes	-	-	1 (0.1%)	3 (0.4%)	13(1.8%)
NR	651 (89.2%)	647 (88.6%)	597 (81.8%)	411 (56.3%)	194 (26.6%)
**Diuretics**					
Thiazide					
No	79 (10.8%)	83 (11.4%)	133 (18.2%)	318 (43.6%)	490 (71.2%)
Yes	-	-	-	1 (0.1%)	4 (0.6%)
NR	651 (89.2%)	647 (88.6%)	597 (81.8%)	411 (56.3%)	194 (28.2%)
Frusemide					

No	36 (5.2%)	40 (5.8%)	90 (13.1%)	276 (40.1%)	531 (72.7%)
Yes	1 (0.1%)	1 (0.1%)	1 (0.1%)	1 (0.1%)	5 (0.7%)
NR	651 (94.6%)	647 (94.0%)	597 (86.8%)	411 (59.7%)	194 (26.6%)
Spironolactone					
No	78 (10.7%)	82 (11.2%)	132 (18.1%)	318 (43.6%)	532 (72.9%)
Yes	1 (0.10%)	1 (0.10%)	1 (0.1%)	1 (0.10%)	4 (0.5%)
NR	651 (94.6%)	647 (88.6%)	597 (81.8%)	411 (56.3%)	194 (26.6%)
**Anticoagulants**					
Aspirin 75					
No	78 (10.7%)	82 (11.2%)	132 (18.1%)	318 (43.6%)	533 (73.0%)
Yes	1 (0.10%)	1 (0.10%)	1 (0.1%)	1 (0.10%)	3 (0.40%)
NR	651 (94.6%)	647 (88.6%)	597 (81.8%)	411 (56.3%)	194 (26.6%)
Clopidogrel 75					
No	74 (10.1%)	79 (10.8%)	127 (17.41%)	311 (42.6%)	520 (71.2%)
Yes	5 (0.70%)	4 (0.50%)	6 (0.8%)	8 (1.10%)	16 (2.20%)
NR	651 (89.6%)	647 (88.6%)	597(81.8%)	411 (56.3%)	194 (26.6%)
**Nitroglycerine**					
No	29 (4.2%)	33 (4.8%)	80 (11.6%)	265 (38.5%)	475 (69.0%)
Yes	7 (1.0%)	7 (1.0%)	10 (1.5%)	12 (1.7%)	19 (2.8%)
NR	652 (94.8%)	648 (94.3%)	598 (86.9%)	411 (59.7%)	194 (28.2%)

NB: NR = Not Recorded.

Table S5 - Details of lipid-lowering medicines prescribed.

	Visit-1	Visit-2	Visit-3	Visit-4	Visit-5
**Statins (and dose)10/20/40mg**					
No	55 (7.50%)	55 (7.5%)	56 (7.7%)	67 (9.2%)	76 (10.4%)
Yes	12 (1.60%)	11 (1.5%)	43 (5.9%)	158 (21.6%)	361 (49.5%)
NR	663 (90.8%)	664 (91.0%)	631 (86.4%)	505 (69.2%)	293 (40.1%)
**Fibrates (and dose)145/200mg**					
No	67 (9.2%)	66 (9.0%)	97 (13.3%)	214 (29.3%)	417 (57.1%)
Yes	1 (0.10%)	-	2 (0.30%)	11 (1.5%)	20 (2.70%)
NR	662 (90.7%)	664 (91.0%)	631 (86.4%)	505 (69.2%)	293 (40.1%)

NB: NR = Not Recorded

Table S6 - Details of micro- and macro-vascular complication rates among the studied patients.

	Visit-1	Visit-2	Visit-3	Visit-4	Visit-5
**Microvascular Complications**					
Retinopathy					
No	43 (6.3%)	48 (6.6%)	106 (14.5%)	345 (47.3%)	611 (83.7%)
Yes	-	-	2 (0.3%)	2 (0.3%)	26 (3.6%)
NR	687 (94.1%)	682 (93.4%)	622 (85.2%)	383 (52.5%)	93 (12.7%)
Nephropathy					
No	43 (6.3%)	47 (6.4%)	107 (14.7%)	328 (44.9%)	602 (82.5%)
Yes	-	1 (0.10%)	1 (0.10%)	19 (2.6%)	35 (4.80%)
NR	687 (94.1%)	640 (93.4%)	622 (85.2%)	383 (52.5%)	93 (12.7%)
Neuropathy					
No	43 (6.3%)	47 (6.4%)	104 (14.2%)	325 (44.5%)	553 (75.8%)
Yes	-	1 (0.10%)	4 (0.50%)	22 (3.0%)	84 (11.5%)
NR	687 (94.1%)	682 (93.4%)	622 (85.2%)	383 (52.5%)	93 (12.7%)
**Macrovascular Complications**					
Cardiovascular disease					
No	39 (5.30%)	43 (5.9%)	101 (13.8%)	323 (44.2%)	565 (77.4%)
Yes	4 (0.50%)	5 (0.7%)	7 (1.0%)	24 (3.3%)	68 (9.3%)
NR	687 (94.1%)	682 (93.4%)	622 (85.2%)	383 (52.5%)	97 (13.3%)
Cerebrovascular disease (Stroke)					
No	43 (5.90%)	48 (6.6%)	106 (14.5%)	335 (45.9%)	616 (84.4%)
Yes	-	-	2 (0.30%)	13 (1.80%)	21 (2.9%)
NR	687 (94.1%)	682 (93.4%)	622 (85.2%)	382 (52.3%)	93 (12.7%)
Peripheral arterial disease					
No	43 (5.90%)	48 (6.6%)	108 (14.8%)	347 (47.5%)	630 (86.3%)
Yes	-	-	-	1 (0.10%)	7 (1.0%)
NR	687 (94.1%)	682 (93.4%)	622 (85.2%)	382 (52.3%)	93 (12.7%)
Diabetic foot					
No	43 (6.3%)	48 (7.0%)	106 (14.5%)	342 (46.8%)	615 (84.2%)
Yes	-	-	2 (0.30%)	6 (0.80%)	22 (3.0%)
NR	687 (94.1%)	682 (93.4%)	622 (85.2%)	382 (52.3%)	93 (12.7%)

NB: NR = Not Recorded

Table S7. Co-morbidities seen among the studied population.

	Visit-1	Visit-2	Visit-3	Visit-4	Visit-5
**Asthma**					
No	43 (6.3%)	48 (7.0%)	107 (14.7%)	347 (47.5%)	625 (85.6%)
Yes	-	-	1 (0.10%)	1 (0.10%)	12 (1.6%)
NR	687 (94.1%)	682 (93.4%)	622 (85.2%)	382 (52.3%)	93 (12.7%)
**COPD**					
No	43 (6.3%)	48 (6.6%)	108 (14.8%)	346 (47.4%)	632 (86.6%)
Yes	-	-	-	2 (0.30%)	4 (0.50%)
NR	687 (94.1%)	682 (93.4%)	622 (85.2%)	382 (52.3%)	93 (12.7%)
**Hypothyroidism**					
No	42 (5.80%)	46 (6.3%)	92 (12.6%)	288 (39.5)	517 (70.8%)
Yes	1 (0.1%)	2 (0.30%)	16 (2.20%)	60 (8.20%)	120 (16.4%)
NR	687 (94.1%)	682 (93.4%)	622 (85.2%)	382 (52.3%)	93 (12.7%)
**Hyperthyroidism**					
No	43 (6.3%)	48 (6.6%)	107 (14.7%)	344 (47.1%)	625 (85.6%)
Yes	-	-	1 (0.10%)	4 (0.50%)	12 (1.6%)
NR	687 (94.1%)	682 (93.4%)	622 (85.2%)	382 (52.3%)	93 (12.7%)

NB: NR = Not Recorded

Table S8 - Other complications and key events.

	Visit-1	Visit-2	Visit-3	Visit-4	Visit-5
**Infection (not specified)**					
Yes	18 (6.7%)	3 (0.4%)	2 (0.3%)	8 (1.1%)	78 (10.7%)
No	49 (6.7%)	19 (2.6%)	77 (10.5%)	293 (40.1%)	626 (85.8)
Not recorded	663 (90.8%)	708 (97.0%)	651 (89.2%)	429 (58.8%)	26 (3.6%)
**Waiting for surgery (surgery not specified)**					
Yes	1 (0.1%)	3 (0.4%)	4 (0.5%)	2 (0.3%)	44 (6.0%)
No	66 (9.0%)	19 (2.6%)	75 (10.3%)	299 (41.0%)	660 (90.4%)
Not recorded	663 (90.8%)	708 (98.9%)	651 (89.2%)	429 (58.8%)	26 (3.6%)
**Required Hospitalization (any medical or surgical cause)**					
No	82 (11.2%)	81 (11.1%)	82 (11.2%)	87 (11.9%)	112 (15.3%)
Yes	6 (0.80%)	5 (0.7%)	9 (1.20%)	17 (2.30%)	138 (18.9%)
NR	642 (87.9%)	644 (88.2%)	639 (87.5%)	626 (85.8%)	480 (65.8%)
**Other Illness including other co-morbidities**					
Not recorded	663 (96.2%)	666 (96.7%)	609 (88.4%)	389 (56.5%)	2 (0.3%)

NB: NR = Not Recorded
